# Prognostic Value of Metabolic Tumor Volume Measured by 18F-FDG PET/CT in Esophageal Cancer Patients

**DOI:** 10.4274/Mirt.07379

**Published:** 2014-02-05

**Authors:** Çiğdem Soydal, Cabir Yüksel, Nuriye Özlem Küçük, İlker Ökten, Elgin Özkan, Beyza Doğanay Erdoğan

**Affiliations:** 1 Ankara University Medical School, Department of Nuclear Medicine, Ankara, Turkey; 2 Ankara University Medical School, Department of Thoracic Surgery, Ankara, Turkey; 3 Ankara University Medical School, Department of Biostatistics, Ankara, Turkey

**Keywords:** Esophageal cancer, positron-emission tomography/computed tomography, tumor volume

## Abstract

**Objective**: In this study, we aimed to explore prognostic importance of definition of preoperative metabolic tumor volume in esophageal cancer patients.

**Methods**: 22 patients who have histologically proven stage IIA-III esophageal cancer and underwent ^18^F-FDG PET/CT for preoperative staging of disease were included to the study. After ^18^F-FDG PET/CT, all the patients underwent surgery within 4 weeks period. Patients have been followed up until death or Sept 15^th^, 2012. Dates of death were recorded for survival analysis. During evaluation of ^18^F-FDG PET/CT images, metabolic tumor volumes were calculated by drawing the isocontour region of interests from all visually positive FGD uptake lesions.

**Results**: 22 patients (15M, 7F; mean age: 65.1±8.4, min-max:48-80) underwent ^18^F-FDG PET/CT for preoperative staging of esophageal cancer. Preoperative diagnosis was squamous cell and adeno cancer in 17 (%77) and 5 (%23) patients, respectively. Location of primary tumor is distal, proximal and mid-esophagus in 13 (%59), 6 (%27) and 3 (%13) patients, respectively. Primary tumor of all the patients were FDG avid (mean SUV_max_: 18.85±7.0; range: 5.5-35.1). Additionally, ^18^F-FDG uptake was seen in mediastinal lymph nodes in 13 patients (5.45±8.15; range: 2.6-29.9). Mean metabolic tumor volumes of primary esophageal lesions were calculated as 8.77±8.46cm^3^ (range: 2.3-34.2). Mean MTV of lymph nodes was 2.44±1.01cm^3^ (range: 0.4-3.6). Mean total metabolic tumor volume was calculated as 9.99±8.58cm^3^ (range: 2.3-27.3). 10 patients died during 447±121 days follow-up period. Mean survival time was 11.9±1.5 months (95%CI: 8.99-14.74) for entire patient group. Total metabolic tumor volume had a significant effect on survival (p=0.045) according to Cox proportional hazards regression analysis. One unit increase in MTV caused 1.1 (95%CI:1.003-1.196) fold increase in hazard, at any time.

**Conclusion**: Definition of preoperative metabolic tumor volume has a prognostic value in the prediction of postoperative survival times. Patients who have higher preoperative metabolic tumor volumes could be good candidates for more aggressive chemo-radiation therapy regiments.

**Conflict of interest:**None declared.

## Abstract (tr)

**Amaç**: Bu çalışmada özofageal kanserli hastalarda preoperatif metabolik tümör volümü hesaplanmasının prognostik önemini araştırmayı amaçladık.

**Yöntemler**: Çalışmaya histopatolojik olarak doğrulanmış ve preoperatif evreleme amacı ile ^18^F-FDG PET/BT uygulanmış evre IIA-III özofagus kanserli 22 hasta dahil edildi. ^18^F-FDG PET/BT’den sonra tüm hastalara 4 haftalık periyod içerisinde cerrahi girişim uygulandı. Hastalar ölüme ya da çalışmanın sonlandırıldığı 15 Eylül 2012 tarihine kadar takip edildi. Ölüm tarihleri sağkalım analizinde kullanılmak üzere kaydedildi. ^18^F-FDG PET/BT görüntülerinin değerlendirilmesi sırasında vizüel olarak FDG tutulumu gösteren tüm lezyonların çevresinden izokontur ilgi alanları çizilerek metabolik tümör volümü hesaplandı.

**Bulgular**: Toplam 22 hastaya (15K, 7E; ort. yaş:65,1±8,4, min-maks:48-80) özofageal kanserin preoperatif evrelemesi amacı ile ^18^F-FDG PET/BT yapıldı. 17 (%77) hastanın preoperatif tanısı yassı hücreli kanser iken, 5 (%23) hastanınki adenokanserdi. Tümör lokalizasyonları sırası ile 13 (%59), 6 (%27) ve 3 (%13) hastada distal, proksimal ve orta özofagustu. Tüm hastaların primer tümörlerinde FDG tutulumu izlendi (ort SUV_max_: 18,85±7,0; min-maks: 5,5-35,1). Ek olarak, 13 hastada mediastinal lenf nodlarında (ort SUV_max_: 5,45±8,15; min-maks: 2,6-29,9) FDG tutulumu saptandı. Özofageal lezyonların ortalama metabolik tümör volümü (MTV) 8,77±8,46cm^3^ (min-maks: 2,3-34,2) olarak hesaplandı. Lenf nodlarının ortalama MTV’si 2,44±1,01cm^3^ (min-maks:0,4-3,6)idi. Toplam ortalama metabolik tümör volümü 9,99±8,58cm^3^ (min-maks:2,3-27,3) olarak hesaplandı. 447±121 günlük takip süresinde 10 hasta exitus oldu. Tüm hasta grubunda ortalama sağkalım süresi 11,9±1,5 (95%CI:8,99-14,74) aydı. Cox hazard regresyon analizine göre toplam metabolik tümör volümü sağkalım üzerine anlamlı bir etkiye sahipti (p=0,045). MTV’de bir birimlik artış, risk oranında 1,1 kat (95%CI:1,003-1,196) artışa sebep olmaktaydı.

**Sonuç**: Preoperatif metabolik tümör volümünün hesaplanması postoperatif sağkalım süresini öngörmede prognostik bir değere sahiptir. Bu nedenle daha yüksek preoperatif metabolik tümör volümüne sahip hastalar, daha agresif kemo-radioterapi rejimleri için iyi adaylar olabilirler.

## INTRODUCTION

Esophageal cancer is the eighth most common malignancy and one of the most common causes of cancer related mortality (1). Disease prognosis is strongly related with stage at diagnosis, because for most patients diagnosed at late-stage of disease, 5 years survival has been reported to be less than 20% (2). Resectability and overall prognosis depend on tumor stage and disease extent (3).

Recently, metabolic tumor volume (MTV) measured by ^18^F-FDG PET/CT has been described as a new prognostic factor in several tumors (4,5,6,7). Because of nonhomogeneous metabolic pattern of tumors, definition of metabolic tumor volume could be more valuable than measurement of maximum standardized uptake value (SUV_max_).

In this study, we aimed to explore prognostic importance of preoperative metabolic tumor volume in stage IIA-III esophageal cancer patients.

## MATERIALS AND METHODS

**Patients**

This retrospective study was designed to search esophageal cancer patients who were referred for ^18^F-FDG PET/CT for preoperative staging of disease between February 2011 and April 2012. Patients who have previous neoadjuvant chemotherapy history or inoperable disease were excluded from the study. Thus, 22 patients who have histologically proven stage IIA-III esophageal cancer and underwent ^18^F-FDG PET/CT for preoperative staging of disease were included to the study. After ^18^F-FDG PET/CT, all the patients underwent surgery within 4 weeks period. Patients have been followed up until death or 15^th^ Sept, 2012. Dates of death were recorded for survival analysis.

**^18^F-FDG PET/CT**

PET/CT images were acquired with a GE Discovery ST PET/CT scanner. Patients fasted at least 6 hours before imaging and blood glucose levels were checked. Those with a blood glucose level above 150 mg/dL did not undergo scanning. Oral contrast was given to all patients. Images from the vertex to the proximal femur were obtained while the patients were in the supine position. Whole body ^18^F-FDG PET/CT imaging was performed approximately 1 hour after an intravenous injection of 8-10 mCi 18F-FDG. During the waiting period, patients rested in a quiet room without taking any muscle relaxants. PET images were acquired for 4 minutes per bed position. Emission PET images were reconstructed with noncontrast CT images. CT images were also obtained from the patient’s integrated F^18^-FDG PET/CT with the use of a standardized protocol of 140 kV, 70 mA, tube rotation time of 0.5 s per rotation, a pitch of 6 and a slice thickness of 5 mm. Patients were allowed to breathe normally during the procedure. Attenuation-corrected PET/CT fusion images were reviewed in three planes (transaxial, coronal and sagittal) on a Xeleris Workstation 4.2 (GE Medical Systems). PET/CT images were evaluated and confirmed visually and semi-quantitatively with standardized uptake value (SUV) by consensus of two experienced nuclear medicine specialists. During evaluation of ^18^F-FDG PET/CT images, MTVs were calculated by drawing automatically the isocontour region of interests (ROI) from all visually FDG uptake lesions.

**Surgical Procedures**

After ^18^F-FDG PET/CT, all the patients underwent partial esophagus resection procedures according to localization of primary tumors and regional lymph node dissection within 1-4 weeks period. Adjuvant systemic chemotherapy was given to stage III patients.

**Statistical Analysis**

Data were summarized as mean±standard deviation. Mean survival time and its standard error along with 95% confidence interval (CI) for entire patient group was calculated taking into account censored data information using Kaplan Meier analysis. Cox proportional hazards regression model was conducted to determine the effect of MTVs for survival. A value of p<0.05 was considered significant. All statistical analyses were performed using SPSS computer statistical software (version 15.0; SPSS, Chicago, Illinois). 

## RESULTS

**Patients**

22 patients (15M, 7F; mean age:65.1±8.4, min-max:48-80) underwent ^18^F-FDG PET/CT for preoperative staging of esophageal cancer. Preoperative diagnosis was squamous cell and adeno cancer in 17 (%77) and 5 (%23) patients, respectively. Location of primary tumor is distal, proximal and mid-esophagus in 13 (%59), 6 (%27) and 3 (%13) patients, respectively. None of the patients had taken neo-adjuvant chemotherapy. All the patients had undergone diagnostic thorax CT 1-4 months before ^18^F-FDG PET/CT.

**^18^F-FDG PET/CT**

**Figure 1:**
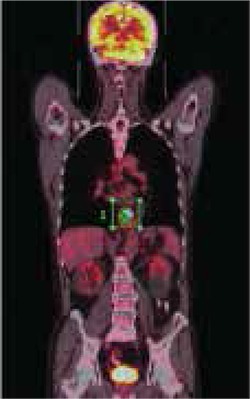
67 year old male patient who had been diagnosed as squa- mous cell cancer by endoscopic biopsy of distal esophageal lesion. 18F-FDG PET/CT showed FDG uptake in distal esophageal lesion with 17.9 SUV_max_ and MTV was calculated as 22.22 cm^3^.

**Figure 2:**
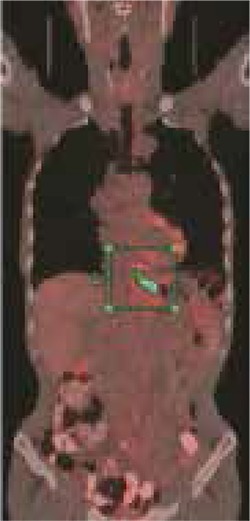
66 year old male patient whose distal esophageal lesion was reported as squamous cell cancer by endoscopic biopsy. 18F-FDG PET/ CT showed FDG uptake in distal esophageal lesion with 14.7 SUV_max_ and MTV was calculated as 23.90 cm^3^.

All the patients underwent ^18^F-FDG PET/CT for preoperative staging of disease. Primary tumor of all the patients were FDG avid (mean SUV_max_: 18.85±7.0; range:5.5-35.1). Additionally, ^18^F-FDG uptake was seen in mediastinal lymph nodes in 13 patients (5.45±8.15; min-max:2.6-29.9). Mean MTV of primary esophageal lesions was calculated as 8.77±8.46cm^3^ (range:2.3-34.2). Mean MTV of lymph nodes was 2.44±1.01cm^3^ (range:0.4-3.6). Total MTV were computed by sum of primary tumor volumes and lymph nodes. Mean total MTV was calculated as 9.99±8.58cm^3^ (range:2.3-27.3). Two examples for calculation of MTVs were demonstrated in Figure 1 and 2.

**Survival**

10 patients died during 447±121 days of follow-up period. Mean survival time was 11.9±1.5 months (95% CI: 8.99-14.74) for entire patient group. Total MTV had significant effect on survival (p=0.045) according to Cox proportional hazards regression analysis. One unit increase in total MTV caused 1.1 (95%CI:1.003-1.196) fold increase in hazard, at any time.

## DISCUSSION

Prediction of disease prognosis and survival in preoperative period is crucial for consideration of more aggressive pre or postoperative adjuvant treatments in selected esophageal cancer patients (8). ^18^F-FDG PET/CT is a metabolic imaging method and its usefulness in esophageal cancer patients has been reported in several studies (9,10,11,12). SUV has been widely used parameter for evaluation of FDG uptake degree of several tumor types. However, because generally tumors have nonhomogeneous ^18^F-FDG uptake pattern, SUV could be a rough parameter in the evaluation of total lesion glucose metabolism.

Recently, new studies focused on evaluation of tumor burden by metabolic tumor volume measurements instead of evaluation of SUV. It is reported that MTV is a prognostic factor for prediction of disease prognosis in several tumors (4,5,6,7). In this study we described the prognostic importance of delineation of preoperative MTV in esophageal cancer patients. We only included operable stage IIA-III patients for standardization of patients. In inoperable advanced stage patients, disease prognosis could be affected by several factors such as different chemo or radiation therapy protocols. Prognostic role of MTV might be explored in inoperable patients who took similar chemoradiation therapy regiments. However in this case delineation of MTVs might be practically impossible in patients who have multiple distant metastases.

Predictive value of ^18^F-FDG PET/CT in the evaluation of regional lymph node metastases has been investigated by Hsu et al. (12). They concluded that SUV_max_ of extra-tumoral uptake and the number of PET abnormalities were significantly associated with N classification. I et al. have reported relationship between MTV and lymph node status in esophageal cancer patients (3). We investigated prognostic value of MTV in esophageal cancer patients. Because lymph node status and disease stage are strongly correlated with disease prognosis, results of our study are concordant with the literature. We found a statistically significant relationship between MTV and survival times. However we could not define a threshold for MTV to predict disease prognosis because of the limited number of patients. Larger and prospective new studies are needed to describe possible threshold for MTV in esophageal cancer. Preliminary results of this study might lead to establish new studies in this area.

## CONCLUSION

Definition of preoperative MTV is a prognostic value in the prediction of postoperative survival times. Patients who have higher preoperative MTVs could be good candidates for more aggressive chemo-radiation therapy regiments.
